# Fever-Germs in Milk

**Published:** 1870-11

**Authors:** 


### Fever-Germs in Milk.

As a pendant to this, we subjoin a letter from Dr. Lawson Tait, to the editor of the *British Medical Journal*, on this subject, as follows:

SIR,—A note in your last issue, on the spread of scarlet fever by milk, leads me to hasten the publication of an observation and some experiments which I made in April last on this subject; and I do so partly that I may have some credit for an original observation, and partly to lead others to observe what I have scarcely time now to work out.

In the month of April last I was engaged, with my friend M. E. Naylor, veterinary surgeon, in examining the conditions attending the spread of the foot-and-mouth disease, in the West Riding; and, amongst other statious of suffering, we visited the farm attached to the West Riding Lunatic Asylum, under the superintendence of my distinguished friend, Dr. Crichton Browne. I had a long conversation with the intelligent farm bailiff, Mr. Turner; and, amongst other experiences, I tasted the diseased milk. I found that this had a peculiarly disagreeable smoky taste, and at first I rashly set this down as due to the disease of the cows. I found, however, that this smoky taint was by no means confined to the milk yielded by the affected animals; and Dr. Browne told me that he had sometimes occasion to send away milk and cream from his table, which was unfit to use on account of this smoky taste. A little examination further showed us that this flavoring was due to the recent asphalting which had been done in and near the milk-house. It at once flashed across my mind that, if milk acquired this tarry flavor from the absorption of the exhalations of asphalting, it was just possible that it might also acquire other things which were not so innoxious; and I at once set going a series of experiments which have led me to the belief that milk is an extremely dangerous agent for the spread of contagion. I need not say that I did not try any experiments, as they were all personal, with contagious matter; but by enclosing fresh milk under bell-jars with tar, turpentine, assafœtida, fæces, urine, etc., I found that in most instances the milk became impregnated with the smell, and sometimes with that intensely disagreeable sensation which we know as the "taste like the smell" of the substances employed. The degree to which this was acquired seemed not so much to be in proportion to the amount employed either of milk or infectant substances, but to the amount and quality of the cream which rose to the surface of the milk; the oleaginous molecules seeming to act as the menstruum of contagion. This is not unlikely, when we remember that the best solvent for nearly all odoriferous principles is oil. Clinically, this question will be most difficult and dangerous to work out. For one I shall not attempt it. But, if we bethink ourselves of any instances of diseases which might in certain instances be communicated by milk, typhoid fever stands out with fearful probability. Enteric fever is nowhere more common nor more fatal than in country farm-houses, where means for the removal of the dejections are not sufficiently well adapted for security, and much too convenient for safety. Epidemics of typhoid fever break out in towns, limited sometimes to a house or particular family, in such way as to defy any explanation by deficiencies of drainage or water supply. I am certain that I have seen cases that might bear the explanation of milk-poisoning, although I have not had the opportunity of working out the facts—facts which, even if worked out, might be capable of other explanations. The question is a difficult one, but worthy of consideration; and the letter in the *Times* of Thursday last, to which you have alluded, as describing the possibility of the spread of the contagion of scarlet fever, by means of milk, strengthens the view I am inclined to hold—that such communication is rather common.

I am, etc,

LAWSON TAIT.—*Med. Gazette.*

